# Prevalence and Associated Risk Factors of African Animal Trypanosomiasis in Cattle in Lambwe, Kenya

**DOI:** 10.1155/2022/5984376

**Published:** 2022-07-14

**Authors:** Ivy Okello, Eliakunda Mafie, Gillian Eastwood, Jahashi Nzalawahe, Leonard E. G. Mboera, Samuel Onyoyo

**Affiliations:** ^1^SACIDS Foundation for One Health, Africa Centre of Excellence for Infectious Diseases of Humans and Animals in East and Southern Africa, P.O. Box 3297, Morogoro, Tanzania; ^2^Department of Microbiology, Parasitology and Biotechnology, Sokoine University of Agriculture, P.O. Box 3019, Chuo Kikuu, Morogoro, Tanzania; ^3^Kenya Tsetse and Trypanosomiasis Eradication Council, P.O. Box 66290-00800, Nairobi, Kenya; ^4^College of Agriculture & Life Sciences, Virginia Polytechnic Institute & State University, Blacksburg, VA, USA; ^5^Biotechnology Research Institute, Kenya Agricultural and Livestock Research Organization, P.O. Box 362, Kikuyu, Kenya

## Abstract

**Background:**

African animal trypanosomiasis (AAT) affects livestock productivity in sub-Saharan Africa. This study aimed to determine cattle AAT's prevalence and associated risk factors in Lambwe Valley, Kenya.

**Methods:**

In a cross-sectional survey, livestock owners were recruited from four villages of Lambwe in Homa Bay, Kenya. Blood samples were collected from the jugular veins of cattle, and buffy coat smears were examined under a microscope. Parasites were further detected using polymerase chain reaction (PCR). Using a semistructured questionnaire, livestock owners were interviewed on their knowledge of AAT and control practices. Chi-square and multilevel models were used for the analysis.

**Results:**

The overall prevalence was 15.63% (71/454). *Trypanosoma vivax* 10.31% and *T. congolense* Savannah 6.01% were the common species and subspecies. A total of 61 livestock keepers were involved in the study. Of these, 91.80% (56/61) knew AAT, and 90.16% (55/61) could describe the symptoms well and knew tsetse fly bite as transmission mode. Self-treatment (54.09%; 33/61) was common, with up to 50.00% of the farmers using drugs frequently. Isometamidium (72.13%; 44/61) and diminazene (54.09%; 33/61) were drugs frequently used. Although 16.39% (10/61) of the farmers claimed to use chemoprophylactic treatment, 6/10 did not use the right drugs. Animals (92.1%; 58/63) with clinical signs had positive infections. Villages closer to the national park recorded a higher prevalence. Infections were higher in cattle owned by those self-treating (27.23%; 58/213), those using drug treatment without vector control (27.62%; 50/181), those using single-drug therapy, and those practicing communal grazing (20.00%; 59/295). Clinical signs strongly associate with positive infections under multilevel modeling.

**Conclusion:**

Cattle trypanosomiasis is prevalent in the Lambwe region of Kenya. This is influenced by inappropriate control practices, communal grazing, and the proximity of farms to the national park. In addition, clinical signs of the disease have a strong association with infections.

## 1. Introduction

Most of sub-Saharan African countries depend on agricultural production, particularly livestock, to sustain their economy [1]. However, despite livestock's important role in the region, their productivity is affected by infectious diseases such as African animal trypanosomiasis (AAT) [1]. AAT is a significant livestock disease in tsetse-infested regions of Africa, resulting in morbidity and mortality-related losses. Morbidity-related losses are characterized by low milk production, increased risk of infection by other diseases, low live weight gain, and reduced fertility, among others [2]. In addition, mortality may occur if an animal is not treated in good time [2]. Estimates show that AAT results in approximately US$ 4.8 billion in economic losses [3], with more than 50 million animals at risk of infection [4]. Livestock species affected by AAT include cattle, goats, sheep, camels, and dogs [5–7]. However, cattle are reported to be the most affected domestic animal [8, 9].

The disease is mainly transmitted cyclically by tsetse flies (*Glossina* spp.), which carry and transmit different species of trypanosomes such as *Trypanosoma vivax*, *T. congolense*, *T. brucei*, *T. simiae*, *T. theileri*, and *T. evansi* which infect domestic animals including cattle [10–12]. *T. congolense*, *T. vivax*, and *T. brucei* are the species considered most common in cattle [5, 13]. Moreover, mechanical transmission by other biting flies like *Tabanids* and *Stomoxys* has been associated with *T. vivax*, *T. evansi*, and *T. theileri* infections [14, 15]. In cattle, the disease presents itself in an acute or chronic state, characterized by anemia, sporadic fever, weight loss, swollen lymph nodes, and eventual death if not treated [10].

AAT diagnosis in cattle in most African countries depends on clinical presentation and microscopic examination of parasites in blood [16]. This however has had some challenges since clinical diagnosis may be confused with other diseases presenting similar clinical signs. Furthermore, microscopic examination is only helpful at detecting parasitemia in the acute phase when parasites are still in the blood and cannot give a good indication of infection at the chronic stage [16]. On the other hand, molecular detection using polymerase chain reaction (PCR) has been used in some African countries [17–19]. This is considered a highly sensitive and specific technique in both phases of AAT [20].

In Kenya, AAT has been reported in several regions such as western Kenya [21], Suba and Teso districts, Busia county [22–24], Homa Bay Lambwe Valley foci [25], and Coast region [26]. *T. vivax* has been reported to be the most detected pathogen in the diagnosis of most cattle [22, 23]. Homa Bay has reported high populations of *Glossina pallidipes* over the years, due to the interlinking of the tsetse belt with South-Eastern Uganda, a region that has experienced reinvasions of the tsetse species [27], despite the control interventions implemented in the region. This has led to the persistence of AAT in the region, with a prevalence of 9.20% being reported by a recent study based on microscopy [25].

Control of AAT in Africa focuses at the vector level using different techniques such as insecticide-treated traps, cattle repellants, and irradiated sterile vectors [28, 29]. However, these methods are expensive to maintain and hence lack consistency [30]. Chemotherapeutic and chemoprophylactic use of trypanocidal drugs targeted at the parasite have also been applied to control AAT. In addition, the use of trypanocidal drugs has been reported among farmers who are most knowledgeable about the disease [31]. However, the major drawback has been drug costs and the emergence of drug-resistant trypanosomes [32]. Studies have implicated poor practices around the use of trypanocidal drugs and the use of nonsanative pairs of the trypanocidals as some of the contributing factors to the development of drug-resistant strains of trypanosomes [33, 34].

One of the agroecological zones found in Homa Bay is a livestock millet zone in Lambwe-Valley located in lower-midland regions. The majority of the farmers in this region are livestock keepers [35]. Since cattle trypanosomiasis is a major disease in the region, most farmers are thought to know the disease and use insecticides and trypanocidals to control the vector and disease. However, little data is available on the current control practices used by livestock keepers. In addition, control strategies used against vectors have borne less success in the region due to the interlinking of the tsetse belt with Southeastern Uganda. This region has experienced the reemergence of *G. pallidipes* [27]. Moreover, the tsetse population in this region has high genetic diversity, which renders them difficult to control using the available vector control strategies. The insecticides used cannot target all the diverse genes in the *Glossina* species [36].

Different factors have been associated with potential risk factors of AAT in African countries. These include sex and age of the animal [37, 38], body condition [39], type of grazing [13], seasons [40], and closeness to the water sources and national parks or reserves for wild animals [41]. For example, in selected regions of Kenya, higher AAT prevalence has been recorded in older animals and those grazed in communal lands [23, 42]. Still and all, few studies on AAT in cattle have reported on associated risk factors.

Understanding how trypanocidals drug use in developing countries and other risk factors influence the occurrence of cattle AAT is critical in informing the most appropriate control strategy and curbing the development of drug resistance. Therefore, this study aimed to determine the prevalence and associated risk factors of AAT in cattle in Lambwe Valley, Kenya.

## 2. Materials and Methods

### 2.1. Study Area

This study was carried out in the Lambwe Valley in Homa Bay, southwestern Kenya.

Homa Bay lies between longitudes 34°-35° East and latitudes 0°15′–0°52′ South. It has an area of land covering 4,267.1 km^2^. Its geographical location is in the Lake Victoria basin tsetse fly belt, which is densely forested with riverbanks and swamps, an ideal condition for tsetse fly infestation [43, 44]. Lambwe Valley is located in the Western part of the Homa Bay, with Ruma the National Park being part of the Valley. Villages in the lower-midland Lambwe region include Kigoto, Kamato, Gendo, Nyatoto, Korlango, and Ogando. Large cattle keeping is majorly practiced in some of these regions. The regions experience long rains between March and May, dry seasons from June to September, and short rains from October to December [35].

### 2.2. Study Design

This cross-sectional study was done at the end of wet season in May 2021.

### 2.3. Sample Size Determination and Sampling

The sample size was determined based on a 9.2% AAT prevalence in a study done in the region in 2015 that had used microscopic diagnosis [25]. An absolute precision of 5.00% and a confidence interval of 95.00% (1.96 values) were used. The formula explained by Thrusfield (clustering sampling design) [45] was used to calculate the sample size.

(1) $$\begin{array}{c} n=\left\{z2\;P\left(1–P\right)\right\}/d2,\\ n^{\prime }=n\left(1\right.+\rho \left(m–1\right). \end{array}$$

where *N* is the sample size, *P* is the expected prevalence of disease, *Z* is 1.96 constant, *d* is the desired precision of 0.05, *m* is the number of units sampled in each cluster (the number of cattle to be sampled in each village was approximate 15), and *ρ* is the intercluster correlation coefficient of 0.15 [46]. The total number of animals to be sampled was approximately 400.

**Figure 1:**
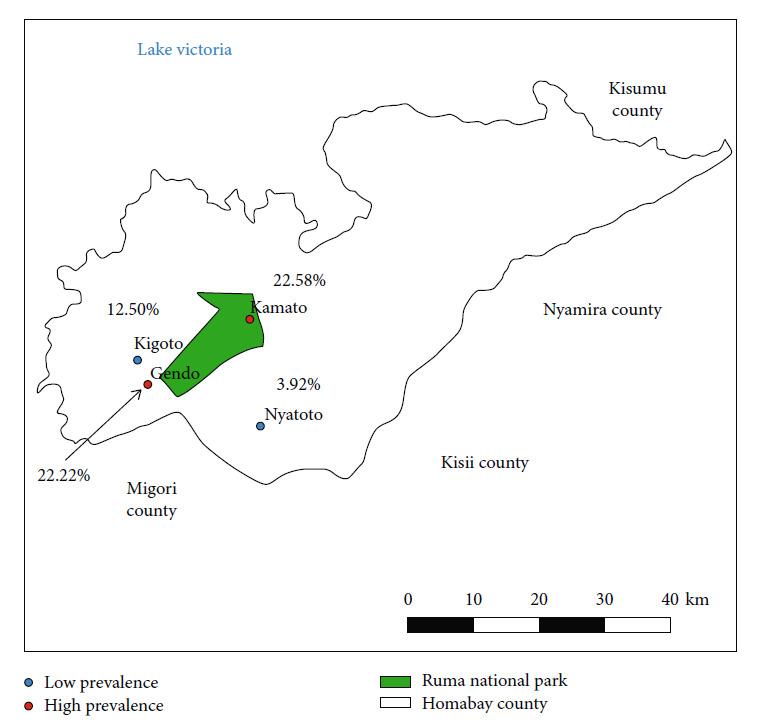
Map of Homa Bay County showing the location of study villages in Lambwe-Valley, with the prevalence detected from each site in percentage.

Sampling was based on villages and herds. Four villages were selected for this study. These were villages located close to Ruma National Park (Figure 1). In addition, these villages had also established large cattle keeping and production systems in the region. The number of animals sampled per village was based on probability proportional to the total animals calculated and the number of villages selected. Animals were then sampled randomly and purposively from different herds belonging to different farmers in each village until the set number for the village was reached (approximately 100-120). A total of 10 animals were randomly sampled in households with many cattle, and an additional one to two cattle were purposively selected if they had clinical signs. Cattle belonging to one household were considered a herd. Therefore, at least 10 households per village were involved. The animals were grouped into two age categories (above five years and below five years). Animals above five years were considered old in this study. Since they are mostly assumed to be more susceptible to diseases due to their low immunity [47–49], we wanted to see if this was also the case for cattle affected by animal trypanosomes in Kenya.

### 2.4. Blood Sample Collection and Processing

Blood samples (5 ml) were collected from the jugular vein of cattle using an 18G needle syringe and stored in BD Vacutainer® blood collection tubes containing potassium ethylenediaminetetraacetic acid (EDTA). Clinical Signs as a level 1 predictor were assessed based on symptoms identified in each cattle, such as weight loss, swollen lymph nodes, low PCV as determined at the field level, and fever. Blood samples were then preserved at 4° Celsius in a cool box containing ice for shipment and storage in a fridge in the laboratory.

Since low packed cell volume (PCV) is a common clinical presentation in cases of AAT [50], both detection of parasites in the buffy coat layer and PCV were determined as described by [50, 51]. A portion of the blood was placed in heparinized capillary tubes, spun at 12,000 rpm, a buffy coat layer extracted, and used to make smears for examination under the microscope at 100 × magnifications. PCV was determined using a hematocrit reader (Hawksley® microhematocrit reader, England).

### 2.5. DNA Extraction and PCR Amplification

DNA extraction was performed for molecular detection of Trypanosoma infection in the lab and followed by conventional PCR machine amplification, using ITS1 and species-specific primers. DNA was extracted from 180 *μ*l whole blood samples using QIAamp DNA Blood Mini kit (Qiagen Inc., Valencia, California, USA). DNA was then stored at -20°C. PCR was then performed on the extracted DNA using the Applied Biosystem Veriti-conventional PCR machine. ITS1 PCR was carried out in a 10 *μ*l reaction containing 1× buffer (1.5 mM MgCl2, 0.2 mM deoxynucleotide triphosphates (dNTPs)), 1 *μ*M ITS1-F and 1 *μ*M ITS1-R, 0.125 U/*μ*l Taq DNA polymerase-MyTaq™ (Bioline, UK), 3.35 *μ*l DNase-free water, and 2.5 *μ*l of template DNA. Primers used were ITS1-F: 5′ CCG GAA GTT CAC CGA TAT TG 3′ and ITS1-R: 5′ TTG CTG CGT TCT TCA ACG AA 3′ [52]. These were used to identify and differentiate trypanosomes by the stretch of their internal transcribed spacer region [52]. The following cycling conditions were used for ITS1 amplification: denaturation at 94°C for 5 min, then 35 cycles of 94°C denaturation for 40 seconds, annealing at 58°C for 40 seconds, and extension at 72°C for 90 seconds, ending with 72°C for 5 minutes. All ITS1-positive samples with 700 bp (*T. congolense*) and 480 bp (*Trypanozoon* species) were subjected to species-specific amplification. Primers were used for *Trypanozoon* subspecies (*Tbb* and SRA) and *T. congolense* subspecies (TCS, TCK, and TCF) (see Table 1). Electrophoresis was then done to the amplified products using a 2% agarose gel (Meridian Bioscience-Bio41026), stained with GelRed (Biotium Hayward, USA); bands were viewed using Gel imager-UVITEC Cambridge.

### 2.6. Interviews with Livestock Keepers

A semistructured questionnaire was administered to 61 livestock keepers, whose cattle were screened for trypanosome infection and had agreed to be part of the questionnaire survey. This was done to assess knowledge, local control practices used by livestock keepers, and risk factors for cattle AAT. Questions asked were on knowledge of AAT on aspects (whether they knew AAT, description of its symptoms, description of how cattle get AAT, how frequent they face the problem, and whether it is a major problem to their cattle, other challenges that face their animals, control practices used (knowledge of control measures, knowledge of trypanocidal drugs, the use and type of trypanocidals used, their source, frequency of use, and person involved in treating the animals) (see Table S1 questionnaire data), and associated risk factors for AAT (type of grazing, closeness of homestead to large water bodies, and national park). Respondents were the household's head/partner of the head. Where the head of the household was missing, the person involved in making decisions for the animals, or was knowledgeable about the animals was selected. A consent form was then signed by each respondent before answering the questions.

### 2.7. Data Analysis

Data were coded and entered into Microsoft Excel. It was then imported to SPSS Version 26 for analysis.

AAT prevalence by site, sex, and age group in animals showing clinical signs and based on species of *Trypanosoma* was analyzed using the chi-square test for descriptive analysis. Descriptive statistics (mean ± SD) were used to present data on packed cell volume (PCV). The mean PCV of infected animals was compared with the mean PCV of noninfected using an independent *t*-test. The chi-square test was also used to determine the association of variables such as local control practices and the observed prevalence cases in cattle.

In addition, a multilevel mixed-effect logistic regression model was used in predicting the probability of a cattle having a positive AAT prevalence as a function of farmer level predictors (knowledge of AAT, symptoms description, use of veterinary prescribed drugs, source of drugs, drug effectiveness, the technique used to control AAT, used drugs, what makes a farmer give the drug to an animal, how frequent they have been using the drug, how close the farm is to national parks/game reserve, what farming system they use, and how close their homestead is to a river/swamp or permanent water body), centered within 4 villages and 61 herds. The following were the model's equations.

Model 1: Intercept only model-Equation (2)

(2) $$\begin{array}{c} {\text{logit}}_{ij}=\beta_{oj},\;\text{Level }1\text{ equation}\\ \begin{array}{cc} \beta_{0j}=\gamma_{00}+\mu_{oj}, & \text{Level }2\text{ equation} \end{array}\\ \begin{array}{cc} {\text{logit}}_{\text{ij}}=\gamma_{00}+\mu_{oj} & \text{Combined equation} \end{array}. \end{array}$$

This was an unconditional model where the study aimed to do modeling between villages and herd variation in logits. The *β*_oj in the level 1 equation represented villages and herd intercept. In this intercept-only model, estimates represented an unconditional village mean and herd mean of the animals (cattle) logits. The level 2 equations contained two components. The *γ*_00 was the only fixed effect in the model. It was the grand mean of the village and herds' means. The *μ*_oj was the difference between the villages and herds *j*'s intercept and the grand mean. There were two parameters estimated in this model: *γ*_00 (grand mean of village and herds means) and ⟦*δ*^2⟧_(*μ*_*oj*) (variance of village intercepts and variance of herds or farmers intercepts). Results were used to determine whether there was significant nonindependence within groups (positive or negative) on the overall prevalence of the disease.

Model 2: level 1 and level 2 fixed predictors' added-Equation (3)

(3) $$\begin{array}{c} \begin{array}{cc} {\text{logit}}_{\text{ij}}=\beta_{\text{oj}}+\gamma_{10}\text{AnimalAge}+\gamma_{20}\text{AnimalSex}, & \text{Combined equation} \end{array}\\ \begin{array}{cc} \beta_{0j}=\gamma_{00}+\gamma_{01}\text{Knowledge}+\gamma_{02}\text{Clinicalsign}+\gamma_{03}\text{Useofveterinary}+\gamma_{04}\text{Howeffective}+\gamma_{05}S\text{ource}+\mu_{oj}, & \text{Level }2\text{ equation } \end{array}\\ \begin{array}{cc} \log it_{ij}=\gamma_{00}+\gamma_{10}\text{AnimalAge}+\gamma_{20}\text{AnimalSex}+\gamma_{01}\text{Knowledg}e+\gamma_{02}\text{Clinicalsign}+\gamma_{03}\text{Useofveterinary}+\gamma_{04}\text{Howeffective}+\gamma_{05}\text{Source}++\mu_{oj}+\mu_{oj} & \text{Combined equation} \end{array}. \end{array}$$

In this model, the prediction was made based on the probability of cattle having a positive prevalence of disease as a function of an animal's level 1 predictor (animals age, sex, clinical signs centered within village and herds) and farmer's level 2 predictors (knowledge of AAT, symptoms described, use of veterinary prescribed drugs, effectiveness, and sources). As a result, there were eight fixed effects (*γ*_00, *γ*_10, *γ*_20, *γ*_02, *γ*_03, *γ*_04, and *γ*_05) and two random effects ⟦*δ*^2⟧_(*μ*_0*j*) and ⟦*δ*^2⟧_(*μ*_1*j*). The parameter to be estimated was the level 2 slope variances.

### 2.8. Ethical Clearance

This study was approved by the Director of Veterinary Services (DVS) in Kenya Ref: MOALF/SDL/DVS/DS/RES/77, National Commission for Science Technology and Innovation (NACOSTI) Ref No: 236117, consent from the farmers and Sokoine University of Agriculture (Ref: SUA/ADM/R.1).

## 3. Results

### 3.1. Trypanosoma Prevalence

A total of 454 cattle were screened for trypanosome infections using microhematocrit buffy coat and molecular techniques. The overall prevalence of buffy coat results was 3.30% (95% CI: 2.21-5.71%) (15/454). On the other hand, molecular ITS1 and species-specific PCR gave 15.63% (95% CI: 12.59-19.27%) (71/454).

**Figure 2:**
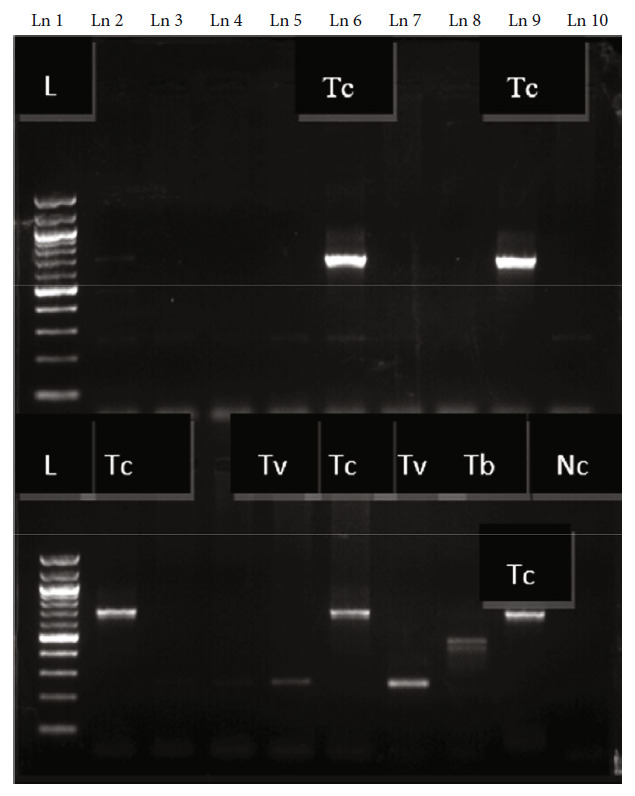
ITS1 PCR bands from one region. Ln stands for lane. Row 1: Ln 1, 100 bp ladder; Ln 2, weak mixed infection; Ln 3, 4, 5, 10, weak *T. vivax*; Ln 6, *T. congolense* and weak *T. vivax*; Ln 9, *T. congolense*. Row 2: Ln 1, ladder; Ln 2 and 6, *T. congolense*; Ln 3, 4, 5, weak *T. vivax*; Ln 7, 8, 9, positive controls; *T. v*, *T. b*, *T. c*, Ln 10, negative control.

*T. vivax* was the most prevalent species (see Figure 2). *T. congolense* followed (see Table 2). All positive samples for *T. congolense* were positive in species-specific results. *T. congolense* Savannah was the most common subspecies, while *T. congolense* Forest and *T. congolense* Kilifi were detected at 1.78% (8/454) and 1.12% (5/454). There were no *T. brucei rhodesiense* or *T. brucei brucei* detected. Mean PCV varied significantly in the different sites. Mean PCV was lower in AAT-positive samples, 24.62 ± 5.483, and higher in the negative samples, 27.03 ± 5.370 (*P* < 0.05).

### 3.2. Demographic Characteristics of the Respondents

A total of 61 farmers were involved in the study. Males accounted for most of the respondents (90.20%; *n* = 55). Most respondents (45.90%; *n* = 28) were between 20 and 40 years old. Those who had primary school education (67.21%; *n* = 41) accounted for most respondents.

### 3.3. Characteristics of Cattle and Farmers' Responses

Female cattle represented slightly over half (52.2%; *n* = 237/454) of the animals sampled. Clinical signs were seen in 73/454 (16.08%) of the animals sampled. Most livestock keepers (91.80%; *n* = 56/61) reported knowing AAT, while only 8.20% (*n* = 5/61) were unaware. Out of those who knew the disease, 90.16% (55/61) could correctly describe the symptoms, with farmers mentioning more than one symptom. Symptoms descriptions for AAT were categorized into two variables. Those with frequency six and above were classified as the “major.” Those with a frequency of less than six were classified as “others.” The “major” symptoms described were weight loss, fever, and death. “Others” symptoms described included pale eyes, abortion, loss of appetite, loss of tail hair, and skin lesions. The majority of livestock keepers (90.16%; *n* = 55/61) also knew how cattle get AAT and described the vector (tsetse fly) mode of transmission accurately (see Table S1 questionnaire data). The same percentage of farmers claimed to know how to control AAT, and out of these, equal proportions of 28/61 used both vector control and drug treatment, and drug treatment solely, while only 4/61 used vector control alone. The most used vector control method was insecticide spraying of cattle. Most livestock keepers mentioned that more than one disease affects their livestock. AAT (93.40%; *n* = 57/61) and Anthrax (21.30%; *n* = 13/61) were the challenges facing most their cattle. Other diseases mentioned were tick diseases, foot and mouth disease (FMD), pneumonia, and worms.

Self-treatment (54.09%; *n* = 33/61) was a common practice, with up to 50.00% of farmers using the drugs frequently (4-8 times/year/animal or 9-above times/year/animal). Isometamidium (72.13%; *n* = 44/61) and diminazene (54.09%; *n* = 33/61) were the most used drugs, followed by homidium chloride. Again, farmers reported using more than one drug. Most drugs were used for chemotherapy, with isometamidium (65.57%; *n* = 40/61) being the most commonly used and homidium chloride (18.03%; *n* = 11/61) the least used. Only ten farmers claimed to be using drugs for prophylaxis, with diminazene (4.67%; *n* = 6/61) the mostly used.

### 3.4. Prevalence of AAT and Its Association to Predictors

Male cattle 18.43% (95% CI: 13.84-24.13%) were more infected than females 13.08% (95% CI: 9.37-17.97%) (31/454). Cattle aged >5 years had more infections 16.67% (95% CI: 12.81-21.40%) (48/454) than cattle <5 years 13.86% (95% CI: 9.41-19.93%) (23/454). However, there was no statistical significance (*P* > 0.05) in the age and sex comparisons (see Table S2), showing the association between overall prevalence and animal predictors based on chi-square descriptive analysis. Animals (92.1%; *n* = 58/63) had clinical signs and were also positive for AAT, while (99.3%; *n* = 292/294) did not have clinical signs and were negative for disease (*P* < 0.05). Villages that were closer to the national park recorded a higher prevalence (see Figure 1) compared to those that were moderately far (*P* < 0.05). In addition, farmers that claimed to live very close to a river/swamp/permanent water body had more positive animals (29.80%; *n* = 28/94) compared to those living close (21.30%; *n* = 17/80) and not close (8.20%; *n* = 15/182) to river (*P* < 0.05).

### 3.5. Local Control Practices and Their Association to Cattle Positive AAT Levels

Nyatoto village had a lower prevalence (3.92%). Respondents (75.00%; *n* = 6/8) who participated in the interview reported using veterinary prescribed drugs or calling veterinarians to treat their animals. Villages such as Gendo and Kamato had higher AAT-positive cases, and most farmers who took part in the interview were self-treating at 66.67% (*n* = 10/15) and 80.00% (*n* = 12/15), respectively. Most who self-treated used single drugs solely (82.76%; *n* = 24/29) or used drugs over frequently (96.88%; *n* = 31/32). Moreover, AAT was higher (27.23%; *n* = 58/213) in cattle owned by respondents who reported self-treating their animals than in those whose animals were treated by a veterinary doctor (1.4%; *n* = 2/142) (*P* < 0.05) (see Table S3), showing the association between prevalence and farmers predictor based on chi-square descriptive statistics. Those who reported treating their animals 4-8 times/year/animal and ≥ 9 times/year/animal had infections detected at 20.61% (*n* = 34/165) and 23.01% (*n* = 26/130), respectively. However, those treated 1-3 times/year/animal did not record any infected animals (*P* < 0.05).

Farmers who gave their animals drug treatment without vector control interventions had (27.62%; *n* = 50/181) positive cattle. On the other hand, those who practiced both vector control interventions and drug treatment had only (4.44%; *n* = 6/136) positive cases (*P* < 0.05). Most positive cases were observed from herds of farmers who used single-drug therapy such as diminazene (23.90%; *n* = 28/117), isometamidium (22.80%; *n* = 18/79), and homidium (37.50%; *n* = 6/16), than from those who used three drugs combination therapy (14.28%; *n* = 1/7) (*P* < 0.05). Only 16.39% (10/61) of the farmers claimed to use chemoprophylactic treatment. However, out of these, 6/10 (diminazene) and 1/10 (homidium) did not use the right drugs indicated for prophylactic use by manufacturers. Only 4/10 used the right chemoprophylactic drug (isometamidium). Farmers who said the drugs worked moderately, not effectively and effectively, had 22.40% (*n* = 15/66), 20.80% (*n* = 32/153), and 9.60% (*n* = 13/135) infected animals, respectively (*P* < 0.05), considering most farmers (98.36%;*n* = 60/61) could correctly describe the symptoms of AAT. Infected animals in farmers that practiced zero grazing were (1.60%; n = 1/63) compared to those who practiced communal grazing (20.00%; 59/295). However, only ten farmers practiced zero-grazing.

### 3.6. Factors that Influenced the Prevalence under a Multilevel Model

The multilevel mixed-effect model was fit, and the target variable was overall prevalence (0 = negative and 1 positive). The probability distribution was binomial (*n* = 454, *P* = 0.156), and the link function was logit. The Akaike and Bayesian information criteria were 1862.165 and 1869.714, respectively. The prediction for the factors (level 1 predictors and level 2 predictors) that influenced the overall prevalence were determined as a function of fixed and random effects. A total of 97 animals were missing in level 2 predictors since there were farmers whose cattle were sampled but did not participate in the interview. The random effect (variance) for 454 animals in 61 herds and four villages was 5.428E-7 and 7.083E-7, respectively. The random effects were minor in level 1 and level 2 predictors. Hence, the model was fit with small variability.

Results indicate that cattle identified with clinical signs had a higher chance of having a positive prevalence than those not. The odds ratio was 316.732, statistically significant at a 5.00% level (*P* value = 0.0001) (Table S4), showing factors influencing prevalence as determined by multilevel mixed effect modeling. This correlation was also observed in descriptive analysis (see Table S2). Hence, clinical signs were the major factor that affected positive AAT prevalence in cattle as a function of herds and villages. On the other hand, other predictors described by the farmers did not have a strong positive influence on prevalence in cattle.

## 4. Discussion

This study revealed that AAT prevalence is still high in Lambwe Valley in Homa Bay, Kenya. The high prevalence reported can be attributed to the densely forested riverbanks and farming activity near the Ruma national park, which has wild animals that can act as reservoirs for animal trypanosomes [55]. Similar findings have been reported in Tanzania in the Maasai Steppe, where cattle kept close to Tarangire National Park and were more exposed to the risk of acquiring AAT [38]. Another study in Uganda also reported more AAT cases in cattle and goats reared closer to Kibale National Park [56]. The high positive cases can also be attributed to farmers' inappropriate local control practices, as seen in this study. The season when the cattle blood samples were collected (late wet season) is also known to have high levels of infected domestic animals [57, 58]. During this season, tsetse flies, livestock, and wild animals are widely spread, increasing chances of contact and transmission. A similar report was given in studies in Nigeria and Tanzania [57, 58]. The use of molecular PCR methods in this study, considered a highly sensitive method compared to microscopy diagnosis that can only detect active parasites in the blood [59], rendered the high detection rates. PCR is also highly particular and can detect different subspecies of trypanosomes in the host based on the primers used [17, 54]. This has also been reported in Zambia and South Africa, where AAT studies have integrated different diagnostic techniques [60–62].

*T. vivax* was the most prevalent species in Lambwe Valley because it can be transmitted via cyclic and mechanical transmission and has a simple lifecycle [63–66]. *T. congolense* was also a common species. Our suggestion is that it can also be transmitted mechanically by *Tabanids* and *Stomoxys* [67, 68]. Hence, this suggests the possible presence of non-tsetse biting flies in the study area, which most farmers could not identify. The most common subgroup of *T. congolense* was *T. congolense* Savannah. The subgroup could be associated with the agroecological condition found in Lambwe. Similar results have been reported in Togo [69]. The Savannah subgroup is also the most harmful strain of the *T. congolense* subgroups leading to a high mortality rate in the animals [70].

AAT levels varied in the studied villages. It is likely due to the different control practices applied. Villages with lower prevalence, such as Nyatoto, had more farmers using veterinary-prescribed drugs, and it is located closer to the larger city. Thus, they had easy accessibility to the veterinary services and drug stores compared to those further away. However, it is also important to note that Nyatoto had fewer farmers participating in the interview. Similar reports were given in a study in Western Uganda, where livestock keepers who provided veterinary services to their animals had fewer cases of *Trypanosoma* infections in their animals [56]. In addition, the proximity of some villages to the national parks and a large water source such as rivers were associated with increased cattle AAT cases. Large water sources and specific agroecological conditions such as densely forested regions are some of the ultimate conditions for tsetse fly infestation [43, 44]. Mean PCV was lower in AAT-positive samples, which was expected as AAT causes anemia, leading to low PCV in animals [10].

Based on the questionnaire results, AAT was the most common disease affecting livestock in the Lambwe region. This may be due to the high abundance of tsetse flies experienced in the region. Farmers who reported the drugs to work moderately and not effectively had a larger proportion of infected animals, considering that most could correctly identify AAT symptoms. The ineffectiveness of drugs is also likely to be associated with inappropriate dosage, inappropriate drug administration, drug quality (and drug storage), and drug resistance [31, 71]. This study could not support some of these findings. However, there were cases of farmers reporting buying trypanocidal drugs from local unlicensed stores, which could affect the quality and storage of drugs.

Using the wrong prophylactic drugs, such as homidium and diminazene, and not treating the animals with chemotherapeutic drugs once they were detected as sick may have led to the observed number of positive cases, despite the prophylactic treatment being highly recommended. Similar findings were seen in a study in North-east Tanzania [72]. Nonetheless, in this study, use of either prophylactic or therapeutic treatment did not seem to have a strong association with the AAT-positive levels. Those who used combination drug therapy had fewer cases of the disease than those who used single-drug therapy. Single-use trypanocidals may lead to ineffective treatment, increasing resistance chances [34]. The high level of reported self-treatment in the study area was associated with the overuse and use of single trypanocidal drugs, which in this case resulted in more positive cases in cattle. This could be due to the selection of resistant strains of trypanosomes. Similar findings have been reported in Togo, where villages with very high drug use had high cases of drug-resistant parasites [69]. This plays a pivotal role in the increase in AAT prevalence. In addition, those who used both vector control and drug treatment had fewer cases of AAT than those who only used drug treatment. It has been suggested that treating cattle population with insecticide leads to increased benefit costs, increased killing of tsetse flies, and reduction of *Trypanosoma* species in cattle and cattle trypanosomiasis [73, 74]. Similar results have been reported in Uganda and Tanzania [73, 74]. Thus, the integrated use of trypanocidals and insecticides can effectively reduce cattle AAT levels.

Communal grazed livestock reporting more positive animals, as was seen in this study, have also been reported in Tanzania [40]. Therefore, it could be associated with livestock moving from tsetse-free regions to tsetse-infested areas in search of pasture and water. Hence, increasing the chances of infection.

From the multilevel analysis, only cattle with clinical signs of AAT strongly associated with the positive prevalence. Similar findings were seen in a study in Kwale district in Kenya [31]. However, level 2 predictors on farmers' knowledge of AAT and the control practices used did not strongly influence positive cases. It could be because most of the cattle from this study were negative for the disease (84.60%), affecting the resulting outcome for the multilevel model in level 2 predictors.

## 5. Limitations

The authors accept there are limitations in terms of the numbers of cattle and herds sampled in terms of extrapolating results to Kenya more generally. One needs to be cautious about the reported prevalence numbers, given the sampling design used.

## 6. Conclusion

AAT is still endemic in the Lambwe region of Homa Bay in Kenya. The high AAT levels reported in cattle have been influenced by communal grazing of cattle and the closeness of villages to the national park. Inappropriate control practices used by farmers contribute to the high prevalence of AAT. Some of these practices include self-treatment, which has led to misuse of trypanocidal drugs, use of single instead of multiple drug therapy, and lack of vector control interventions combined with drug treatment. Emphasis should be placed on training farmers on the proper use of the drugs. Proximity and accessibility to veterinary services could also hinder a farmer from using veterinary prescribed drugs. Clinical signs diagnosis of AAT in cattle is one of the factors that should be considered when examining an animal for possible infections, in conjunction with microscopic or molecular diagnosis.

Emphasis should also be placed on improving government policy on properly using trypanocidals and other control practices. Based on these results, identifying the presence of drug-resistant *Trypanosoma* species should be considered to determine whether the poor control practices could have potentiated the emergence of drug-resistant parasites and whether new drugs need be introduced in the region.

## Figures and Tables

**Table 1:** Species-specific primers used for *Trypanosoma* identification.

*Trypanosoma* species	Sequences	Band size	Reference
*Trypanozoon* (*T. brucei* sub-species)	TbbGPI-PLC-R: TGC CAC CGC AAA GTC GTT ATT TCG	324	[53]
*T. b. brucei*	TbbGPI-PLC-R: CGC TTT GTT GAG GAG CTG CAA GCA		
*T. b. rhodesiense*	SRA-A: GACAACAAGTACCTTGGCGC	460	[53]
	SRA-E: TACTGTTGTTGTACCGCCGC		
*T. congolense* Savannah	TCS1: CGAGAACGGGCACTTGCGA	316	[54]
	TCS2: GGACAAACAAATCCCGCACA		
*T. congolense* Kilifi	TCK1: GTGCCCAAATTTGAAGTGAT	294	[54]
	TCK2: ACTCAAAATCGTGCACCTCG		
*T. congolense* Forest	TCF1: GGA CAC GCC AGA AGG TAC TT	350	[54]
	TCF2: GTT CTC GCA CCA AAT CCA AC		

**Table 2:** Prevalence based on different *Trypanosoma* species in different sites.

Positive cases for trypanosomes								Total tested animals
Site	ITS1	*T. v*	*T. c*	*T. c* Savannah	*T. c* Forest	*T. c* Kilifi	*T. c* & *T. v*	
Kigoto	15	11	3	3	0	0	1	120
Nyatoto	4	3	1	1	0	0	0	102
Kamato	28	18	12	12	7	3	2	124
Gendo	24	14	10	11	1	2	3	108
% prevalence & 95 CI	15.81% (95% CI: 12.73-19.48%)	10.31% (95% CI: 7.82-13.48%)	5.72% (95% CI: 3.88-8.32%)	6.01% (95% CI: 4.17-8.61%)	1.78% (95% CI: 0.91-3.48%)	1.12% (95% CI: 0.48-2.59%)	1.33% (95% CI: 0.61-2.88%)	454

CI = confidence interval; *T. c = T. congolense; T. v = T. vivax.*

## Data Availability

The data on prevalence and associated risk factors to cattle AAT to support the findings of this study are included within the article and others in supplementary information file(s).

## Supplementary Materials

Supplementary Materials The following are availabsle as supplementary materials for this study: Table S1, Table S2, Table S3, Table S4, and Table S5.
