# Supplementary material for: Prevalence and Associated Risk Factors of African Animal Trypanosomiasis in Cattle in Lambwe, Kenya
Source: J Parasitol Res. 2022 Jul 14;2022:5984376. doi: 10.1155/2022/5984376 (PMC9303511; doi:10.1155/2022/5984376)
Supplement: Supplementary Materials — The following are availabsle as supplementary materials for this study: Table S1, Table S2, Table S3, Table S4, and Table S5. [file 5984376.f1.docx]

**Table S1**. Questionnaire


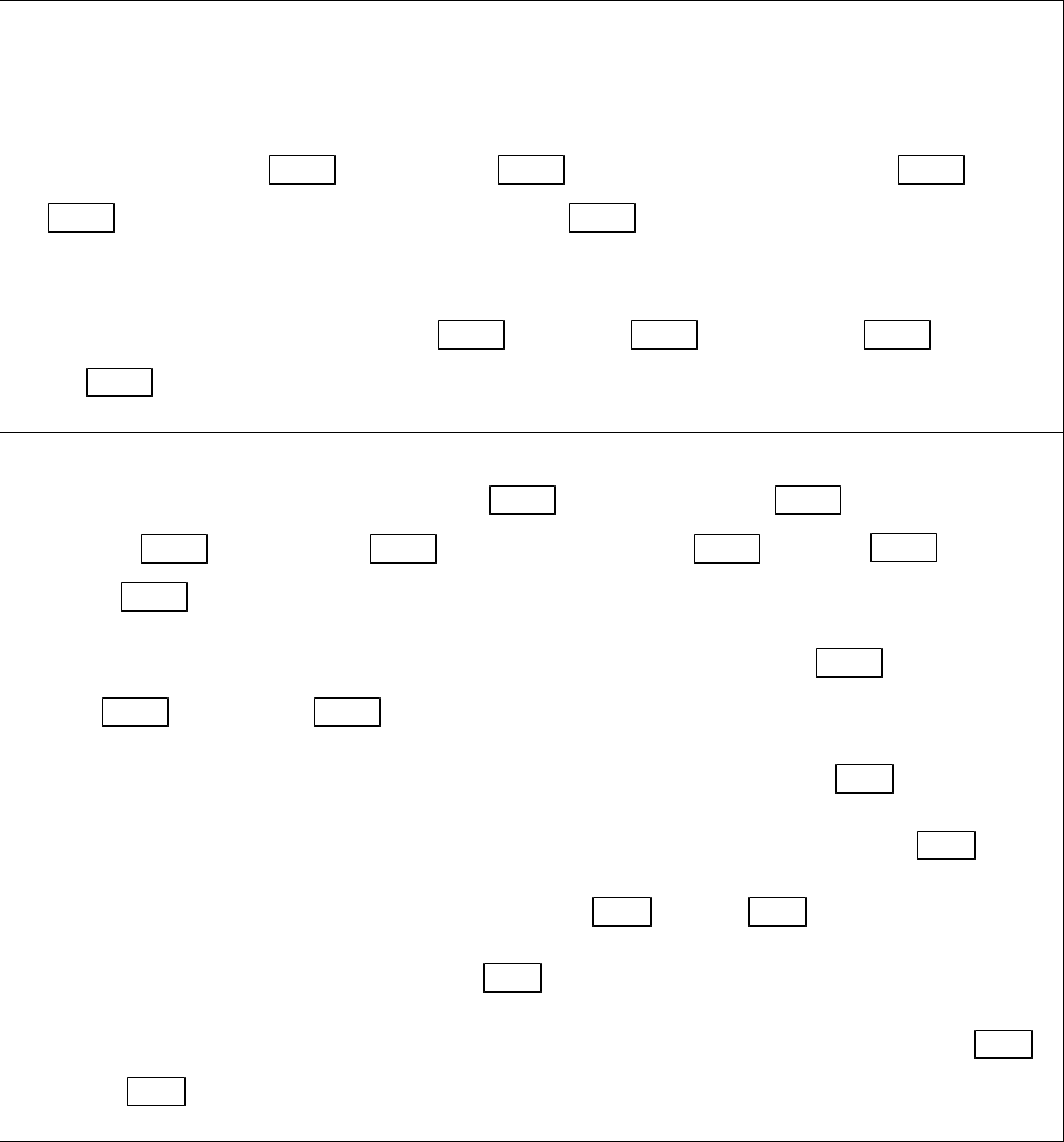


|  |  | **Information about livestock keeper** | |  |  |  |
| --- | --- | --- | --- | --- | --- | --- |
|  |  |  | | |  |  |
|  | **Code:** | **dd/month/year *tarehe siku/mwezi/mwaka*** | | |  |  |
|  |  | |  |  |  |  |
| 1 | Livestock keepers name *majina ya mfugaji* | |  |  |  |  |
|  |  |  |  |  |  |  |
| 2 | Sex female *jinsia mwanamke* | male *mwanamume* | age 20-40years *miaka ishirini mpaka arubaini* | | age 41-60 |  |
|  |  |  |  |  |  |  |
|  | *miaka arubanii mpaka sitini* above 60 years *juu ya miaka sitini* | | |  |  |  |
|  |  |  |  |  |  |  |
| 3 | Village name *jina la kijiji* |  |  |  |  |  |
|  |  |  |  |  |  |  |
| 4 | Level of education *kiwango cha elimu* none *hakuna* | | primary *msingi* | high school *sekondari* | college *chuo* |  |
|  |  |  |  |  |  |  |
|  | *kikuu* |  |  |  |  |  |

|  |  | **Information on the animal** | | |  |  |  |  |  |
| --- | --- | --- | --- | --- | --- | --- | --- | --- | --- |
|  |  |  |  |  |  |  |  |  |  |
| 5 |  |  |  |  |  |  |  |  |  |
| Age of animal <5year *umri wa mnyama chini ya miaka miwili* | | | | >5years *juu ya miaka miwili* | |  | Sex female *jinsia* | |  |
| *mwanamke* | male *mwanamume* | Breed indigenous *mzalishi kienyej*i | | | | exotic *kisasa* | | crossbreed |  |
| *kuzaliana* |  |  |  |  |  |  |  |  |  |
|  |  |  |  |  |  |  |  |  |  |
| 6 |  |  |  |  |  |  |  |  |  |
|  |  |  |  |  |  |  |  |  |  |
| Type of animal *aina ya mnyama* cattle *ng’ombe* | |  | small ruminants goat *wanyama wadogo mbuzi* | | | | sheep |  |  |
| *kondoo* | and pigs *na ngurue* |  |  |  |  |  |  |  |  |
|  |  |  |  |  |  |  |  |  |  |
|  |  |  |  |  |  |  |  |  |  |
| 7 |  |  |  |  |  |  |  |  |  |
| What are the challenges you get from livestock keeping *Unapata changamoto gani kwa ufugaji wa wanyama* | | | | | | |  |  |  |
|  |  |  |  |  |  |  |  |  |  |
| 8 |  |  |  |  |  |  |  |  |  |
| Symptoms description of the disease challenges in your livestock *maelezo ya dalili ya ugonjwa unaosumbua mifugo zako* | | | | | | | |  |  |
|  |  |  |  |  |  |  |  |  |  |
| 9 |  |  |  |  |  |  |  |  |  |
| Do you know African animal trypanosomias (AAT) *unajua nagana* yes/*ndio* | | | | | la/*hapana* |  | if yes, *kama ndio* | |  |
|  |  |  |  |  |  |  |  |  |  |
| 10 |  |  |  |  |  |  |  |  |  |
| What are the symptoms description *ni nini maelezo ya dalili* | | | |  |  |  |  |  |  |
|  |  |  |  |  |  |  |  |  |  |
| 11 |  |  |  |  |  |  |  |  |  |
| Is African animal trypanosomiasis (AAT) a major problem to your animals *nagana ni shida kubwa kwa wanyama wako* yes/*ndio* | | | | | | | | |  |
| la/*hapana* | if yes, *kama ndio* |  |  |  |  |  |  |  |  |



| 12 | How frequent do you face the problem of AAT *unakabiliwa na shida ya nagana mara ngapi* | | | | | | frequently *mara kwa* | |  |
| --- | --- | --- | --- | --- | --- | --- | --- | --- | --- |
|  |  |  |  |  |  |  |  |  |  |
|  | *mara* | not frquently *sio mara kwa mara* | |  |  |  |  |  |  |
|  |  |  |  |  |  |  |  |  |  |
| 13 | Do you know how livestock get AAT *unajua jinsi wanyama wanavyopata nagana* | | | | yes *ndio* | | no *la* |  |  |
|  |  |  |  |  |  |  |  |  |  |
|  |  |  |  |  |  |  |  |  |  |
| 14 | Describe how animals get AAT *eleza jinsi mnyama anapata nagana* | | |  |  |  |  |  |  |
|  |  |  |  |  |  |  |  |  |  |
|  |  |  |  |  |  |  |  |  |  |
| 15 | Do you know how AAT is controlled *unajua nagana inavyodhibitiwa* | | | yes *ndio* |  | no *la* |  |  |  |
|  |  |  |  |  |  |  |  |  |  |
|  |  | | | | | |  |  |  |
| 16 | If yes, what technique do you use to control AAT *kama ndio unatumia njia gani kudhibiti nagana* | | | | | |  |  |  |
|  | Vector control *udhibiti wa vector* | | Traditional *ya jadi* |  | Drug treatment *madawa* | | |  |  |
|  |  |  |  |  |  |  |  |  |  |
| 17 | Do you know trypanocidal drugs *unajua dawa za trypanocidal/nagana* | | | yes *ndio* | | no *la* |  |  |  |
|  |  |  |  |  |  |  |  |  |  |
|  |  |  |  |  |  |  |  |  |  |
| 18 | If yes, do you use trypanocidal drugs *kama ndio unatumiaa dawa za trypanocidal* yes/*ndio* | | | | |  | la/*hapana* | if yes, which |  |
|  |  |  |  |  |  |  |  |  |  |
|  | drug been using *kama ndio unatumia dawa ipi* Berenil *Berenil* | | | veredium *Veridt* | | Bovidium *Bovidium* | |  |  |
|  | Noroquin/Quintrycide/Tribexin *Noroquin/Quintrycide/Tribexin* | | | others *nyegine* | | combination *mchanganyiko* | |  |  |
|  |  | | | | | | | |  |
| 19 | What makes you to give drug to animal *nini kinachokufanya umpe dawa mfugo wako* when it is sick (curative) *akiwa mgonjwa* | | | | | | | |  |
|  | *(tiba)* | for prophylaxis *kwa kuzuia* |  |  |  |  |  |  |  |
|  |  |  |  |  |  |  |  |  |  |
| 20 | How effective the drug is *ufanisi wake* | | effective *inatibu* | not effective *haitibu* | |  | if not effective *kama haitibu* | |  |
|  |  |  |  |  |  |  |  |  |  |
|  |  |  |  |  |  |  |  |  |  |
| 21 | Do you use veterinary prescribed drugs *unatumia dawa iliyoagizwa na daktari wa mifugo* yes/*ndio* | | | | | | la/*hapana* |  |  |
|  |  |  |  |  |  |  |  |  |  |
|  |  |  |  |  |  |  |  |  |  |
| 22 | if yes, from which doctor/which veterinary store *kama ndio, kutoka kwa daktari yupi wa mifugo au duka lipi la mifugo* | | | | | | |  |  |
|  |  |  |  |  |  |  |  |  |  |
|  |  | | | | | | | |  |
| 23 | How frequent have been using the drug 1-3times/year/animal *Ni mara ngapi umetumia dawa; mara1-3/kwa mwaka/kila mnyama* | | | | | | | |  |
|  |  | or 4-8times/year/animal *au mara 4-8/kwa mwaka/kila mnyama* | | | or 9-above times/year/anima *au mara9-juu/kwa* | | | |  |
|  |  |  |  |  |  |  |  |  |  |


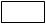

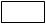

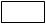

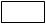

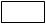

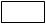

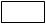

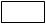

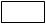

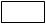

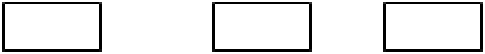

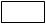

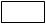

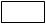

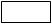

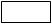

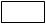

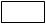

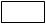

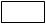

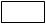

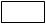

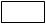

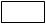

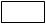

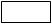

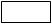

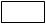

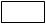


|  | *mwaka/kila mnyama* |  |  |  |  |  |
| --- | --- | --- | --- | --- | --- | --- |
|  |  |  |  | |  |  |
|  |  |  | **Risk factors to AAT** | |  |  |
|  |  | | | | |  |
| 24 | How close is your farm to National Parks/game reserve *ukaribu wa shamba lako na mbuga /hifadhi ya wanyama* very close *karibu sana* | | | | |  |
|  | close *karibu* | not close *sio karibu* | |  |  |  |
|  |  |  |  |  |  |  |
| 25 | What farming system do you use, zero grazing *unatumia malisho gani, sifuri* | | | | communal grazing *malisho ya kijamii* |  |
|  |  |  |  |  |  |  |
|  |  | | | | |  |
| 26 | How close is your homestead to a river/swamp or permanent water body? *Nyumba yako inaukaribu gani na mto/kinamasi/mwili wa maji* | | | | |  |
|  | *wa kudumu* very close *karibu sana* | | close *karibu* | not close *sio karibu* | |  |
|  |  | |  |  |  |  |
|  | **Data collector name and signature:** | |  |  |  |  |
|  |  |  |  |  |  |  |


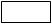

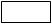

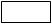

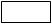

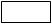

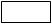

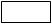

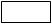

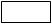


**Table S2.** Association between overall prevalence and animal predictors based on Chi-square descriptive statistics

|  |  |  | Overall | | Prevalence |  |
| --- | --- | --- | --- | --- | --- | --- |
|  |  |  | prevalence | |  |  |
|  |  |  | -ve | +ve | X^2^(P-value) |  |
|  |  |  |  |  |  |  |
|  |  |  |  |  |  |  |
|  | < 5 | Count | 143 | 24 |  |  |
|  |  |  |  |  |  |  |
|  | years | % within Age | 85.60% | 14.40% |  |  |
|  |  | category |  |  |  |  |
| Age |  |  |  |  |  |  |
|  |  |  |  |  | 0.222(0.637) |  |
| category |  | Count | 241 | 46 |  |  |
|  |  |  |  |  |  |  |
|  | > 5 |  |  |  |  |  |
|  |  |  |  |  |  |  |
|  | years | % within Age | 84.00% | 16.00% |  |  |
|  |  | category |  |  |  |  |
|  |  |  |  |  |  |  |
|  |  |  |  |  |  |  |
|  | Female | Count | 207 | 30 |  |  |
|  |  |  |  |  |  |  |
| Sex |  | % within Sex | 87.30% | 12.70% | 2.897(0.089) |  |
|  |  |  |  |  |  |  |
|  |  |  |  |  |  |  |
|  | Male | Count | 177 | 40 |  |  |
|  |  |  |  |  |  |  |
|  |  |  |  |  |  |  |
|  |  | % within Sex | 81.60% | 18.40% |  |  |
|  |  |  |  |  |  |  |
|  |  |  |  |  |  |  |
|  |  | Count | 292 | 2 |  |  |
|  | No |  |  |  |  |  |
|  |  | % within Clinical signs | 99.30% | 0.70% |  |  |
|  |  |  |  |  |  |  |
| Clinical signs |  |  |  |  | 309.871(0.0001) |  |
|  |  | Count | 5 | 58 |  |  |
|  |  |  |  |  |  |  |
|  | Yes |  |  |  |  |  |
|  |  | % within Clinical signs | 7.90% | 92.10% |  |  |
|  |  |  |  |  |  |  |
|  |  |  |  |  |  |  |


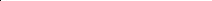

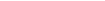

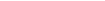

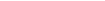

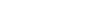

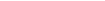

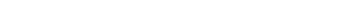

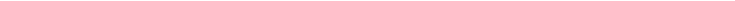

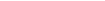

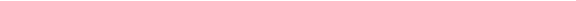

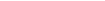


**Table S3**. Association between prevalence and farmers predictor based on Chi-square descriptive statistics

|  |  |  |  |  | Overall prevalence | | | | Prevalence |  |
| --- | --- | --- | --- | --- | --- | --- | --- | --- | --- | --- |
|  |  |  |  |  |  |  |  |  |  |  |
|  |  |  |  |  | -ve |  | +ve | | X^2^(P-value) |  |
|  |  |  |  |  |  |  |  |  |  |  |
|  |  |  |  |  |  |  |  |  |  |  |
| Use of |  |  | Count |  | 142 |  | 2 |  |  |  |
| veterinary |  |  |  |  |  |  |  |  |  |  |
|  | Yes |  | % within Use of |  | 98.60% |  | 1.40% |  |  |  |
| prescribed |  |  |  |  |  |  |  |  |  |  |
|  |  |  | veterinary prescribed |  |  |  |  |  |  |  |
|  |  |  |  |  |  |  |  |  |  |  |
| drugs |  |  |  |  |  |  |  |  |  |  |
|  |  |  | drugs |  |  |  |  |  |  |  |
|  |  |  |  |  |  |  |  |  | 41.032(0.000) |  |
|  |  |  | Count |  | 155 |  | 58 |  |  |  |
|  |  |  |  |  |  |  |  |  |  |  |
|  | Self- |  |  |  |  |  |  |  |  |  |
|  |  |  | % within Use of |  | 72.80% |  | 27.20% |  |  |  |
|  | treatment |  |  |  |  |  |  |  |  |  |
|  |  |  | veterinary prescribed |  |  |  |  |  |  |  |
|  |  |  |  |  |  |  |  |  |  |  |
|  |  |  | drugs |  |  |  |  |  |  |  |
| Source | Agrovet |  | Count |  | 250 |  | 35 |  |  |  |
|  | stores |  |  |  |  |  |  |  |  |  |
|  |  |  | % within Source |  | 87.70% |  | 12.30% |  |  |  |
|  |  |  |  |  |  |  |  |  |  |  |
|  |  |  |  |  |  |  |  |  | 20.703(0.000) |  |
|  | Local |  | Count |  | 47 |  | 25 |  |  |  |
|  |  |  |  |  |  |  |  |  |  |  |
|  | stores |  |  |  |  |  |  |  |  |  |
|  |  |  | % within Source |  | 65.30% |  | 34.70% |  |  |  |
|  |  |  |  |  |  |  |  |  |  |  |
|  |  |  |  |  |  |  |  |  |  |  |
| How | Effective |  | Count |  | 123 |  | 13 |  |  |  |
| effective |  |  |  |  |  |  |  |  |  |  |
|  |  |  | % within How |  | 90.40% |  | 9.60% |  |  |  |
|  |  |  |  |  |  |  |  |  |  |  |
|  |  |  | effective |  |  |  |  |  |  |  |
|  | Not |  | Count |  | 122 |  | 32 |  | 8.340(0.015) |  |
|  | effective |  |  |  |  |  |  |  |  |  |
|  |  |  |  |  |  |  |  |  |  |  |
|  |  |  | % within How |  | 79.20% |  | 20.80% |  |  |  |
|  |  |  |  |  |  |  |  |  |  |  |
|  |  |  | effective |  |  |  |  |  |  |  |
|  | Moderate |  | Count |  | 52 |  | 15 |  |  |  |
|  |  |  |  |  |  |  |  |  |  |  |


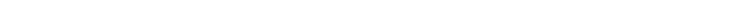

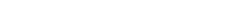

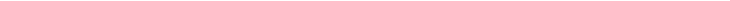

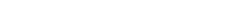

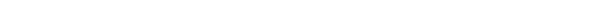

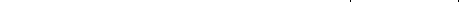

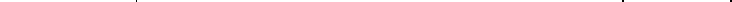

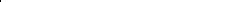

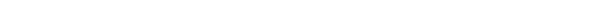

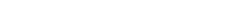

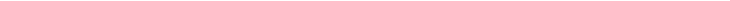

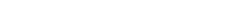

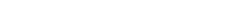

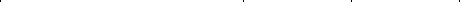

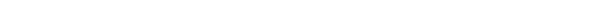


|  |  | % within How | 77.60% | 22.40% |  |  |
| --- | --- | --- | --- | --- | --- | --- |
|  |  | effective |  |  |  |  |
|  | Drugs | Count | 131 | 50 |  |  |
|  |  |  |  |  |  |  |
|  |  | % within What | 72.40% | 27.60% |  |  |
|  |  | technique do you use |  |  |  |  |
|  |  | to control AAT ? |  |  |  |  |
|  | Drugs & | Count | 129 | 6 |  |  |
|  | vector |  |  |  |  |  |
| What |  | % within What | 95.60% | 4.40% |  |  |
|  |  |  |  |  |  |  |
| technique |  | technique do you use |  |  |  |  |
| do you |  | to control AAT ? |  |  | 32.262(0.000) |  |
| use to | Vector | Count | 27 | 4 |  |  |
|  |  |  |  |  |  |  |
| control |  |  |  |  |  |  |
|  |  | % within What | 87.10% | 12.90% |  |  |
| AAT |  |  |  |  |  |  |
|  |  | technique do you use |  |  |  |  |
|  |  |  |  |  |  |  |
|  |  | to control AAT ? |  |  |  |  |
|  | Drug, | Count | 10 | 0 |  |  |
|  | vector & |  |  |  |  |  |
|  |  | % within What | 100.00 | 0.00% |  |  |
|  | tradition |  |  |  |  |  |
|  |  | technique do you use | % |  |  |  |
|  |  |  |  |  |  |  |
|  |  | to control AAT ? |  |  |  |  |
|  | 1-3 | Count | 79 | 0 |  |  |
|  | times/yr/ |  |  |  |  |  |
|  |  | % within How | 100.00 | 0.00% |  |  |
|  | animals |  |  |  |  |  |
|  |  | frequent have been | % |  |  |  |
|  |  |  |  |  |  |  |
|  |  | using the drug |  |  |  |  |
| How | 4-8 | Count | 131 | 34 |  |  |
| frequent |  |  |  |  |  |  |
|  | times/yr/ |  |  |  |  |  |
| have been |  | % within How | 79.40% | 20.60% | 20.772(0.000) |  |
|  | animals |  |  |  |  |  |
|  |  | frequent have been |  |  |  |  |
| using the |  |  |  |  |  |  |
|  |  |  |  |  |  |  |
|  |  | using the drug |  |  |  |  |
| drug |  |  |  |  |  |  |
|  | 9 | Count | 87 | 26 |  |  |
|  | above/tim |  |  |  |  |  |
|  |  | % within How | 77.00% | 23.00% |  |  |
|  | es/yr/ani |  |  |  |  |  |
|  |  | frequent have been |  |  |  |  |
|  | mal |  |  |  |  |  |
|  |  | using the drug |  |  |  |  |
|  |  |  |  |  |  |  |
|  | Close | Count | 107 | 10 |  |  |
|  |  |  |  |  |  |  |
|  |  | % within How Close | 91.50% | 8.50% |  |  |
|  |  | is your farm to |  |  |  |  |
|  |  | National Parks/game |  |  |  |  |
|  |  | reserve |  |  |  |  |
| How Close | Very close | Count | 131 | 45 |  |  |
| is your |  |  |  |  |  |  |
|  |  |  |  |  |  |  |
| farm to |  | % within How Close | 74.40% | 25.60% | 19.074(0.000) |  |
|  |  | is your farm to |  |  |  |  |
| National |  |  |  |  |  |  |
|  |  |  |  |  |  |  |
|  |  | National Parks/game |  |  |  |  |
| Parks/gam |  |  |  |  |  |  |
|  |  | reserve |  |  |  |  |
| e reserve |  |  |  |  |  |  |
|  | Not close | Count | 59 | 5 |  |  |
|  |  |  |  |  |  |  |
|  |  | % within How Close | 92.20% | 7.80% |  |  |
|  |  | is your farm to |  |  |  |  |
|  |  | National Parks/game |  |  |  |  |
|  |  | reserve |  |  |  |  |
|  | Communa | Count | 236 | 59 |  |  |
|  | l |  |  |  |  |  |
|  |  | % within What | 80.00% | 20.00% |  |  |
|  |  |  |  |  |  |  |
| What |  | farming system do |  |  |  |  |
| farming |  | you use ? |  |  | 12.388(0.000) |  |
| system do | Zero | Count | 61 | 1 |  |  |
|  |  |  |  |  |  |  |
| you use |  |  |  |  |  |  |
|  |  | % within What | 98.40% | 1.60% |  |  |
|  |  |  |  |  |  |  |
|  |  | farming system do |  |  |  |  |
|  |  | you use ? |  |  |  |  |
| How Close | Close | Count | 63 | 17 |  |  |
|  |  |  |  |  |  |  |
| is your |  |  |  |  |  |  |
|  |  | % within How Closeis | 78.80% | 21.30% |  |  |
| homestea |  |  |  |  | 22.160(0.000) |  |
|  |  | your homestead to a |  |  |  |  |
| d to a |  |  |  |  |  |  |
|  |  | river/swamp or |  |  |  |  |
| river/swa |  |  |  |  |  |  |
|  |  | permanent water |  |  |  |  |
|  |  |  |  |  |  |  |


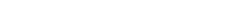

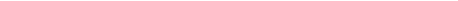

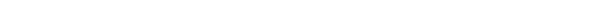

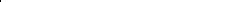

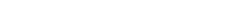

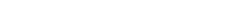

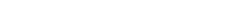

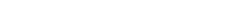

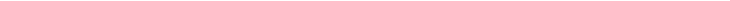

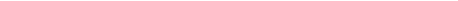

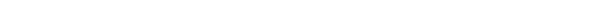

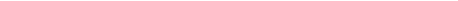

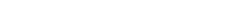

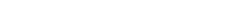

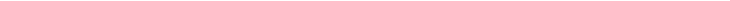

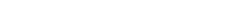

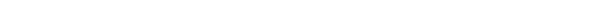


| mp or |  | body? |  |  |  |  |
| --- | --- | --- | --- | --- | --- | --- |
| permanen |  |  |  |  |  |  |
|  | Very close | Count | 66 | 28 |  |  |
| t water |  |  |  |  |  |  |
|  |  |  |  |  |  |  |
| body? |  |  |  |  |  |  |
|  |  | % within How Closeis | 70.20% | 29.80% |  |  |
|  |  | your homestead to a |  |  |  |  |
|  |  | river/swamp or |  |  |  |  |
|  |  | permanent water |  |  |  |  |
|  |  | body? |  |  |  |  |
|  | Not close | Count | 168 | 15 |  |  |
|  |  |  |  |  |  |  |
|  |  | % within How Closeis | 91.80% | 8.20% |  |  |
|  |  | your homestead to a |  |  |  |  |
|  |  | river/swamp or |  |  |  |  |
|  |  | permanent water |  |  |  |  |
|  |  | body? |  |  |  |  |
|  | No | Count | 89 | 9 |  |  |
|  |  |  |  |  |  |  |
|  |  | % within fever | 90.80% | 9.20% |  |  |
| fever |  |  |  |  | 5.614(0.018) |  |
|  | Yes | Count | 208 | 51 |  |  |
|  |  |  |  |  |  |  |
|  |  |  |  |  |  |  |
|  |  | % within fever | 80.30% | 19.70% |  |  |
|  |  |  |  |  |  |  |
|  | No | Count | 66 | 4 |  |  |
|  |  |  |  |  |  |  |
| weight |  | % within weight loss | 94.30% | 5.70% |  |  |
|  |  |  |  |  | 7.662(0.006) |  |
| loss | Yes | Count | 231 | 56 |  |  |
|  |  |  |  |  |  |  |
|  |  |  |  |  |  |  |
|  |  | % within weight loss | 80.50% | 19.50% |  |  |
|  |  |  |  |  |  |  |
| mortality | No | Count | 148 | 34 |  |  |
|  |  |  |  |  |  |  |
|  |  | % within mortality | 81.30% | 18.70% |  |  |
|  |  |  |  |  | 0.933(0.334) |  |
|  | Yes | Count | 149 | 26 |  |  |
|  |  |  |  |  |  |  |
|  |  |  |  |  |  |  |
|  |  | % within mortality | 85.10% | 14.90% |  |  |
|  |  |  |  |  |  |  |
| Skin lesion | No | Count | 294 | 57 |  |  |
|  |  |  |  |  |  |  |
|  |  | % within Skin lesion | 83.80% | 16.20% |  |  |
|  |  |  |  |  | 4.809(0.028) |  |
|  | Yes | Count | 3 | 3 |  |  |
|  |  |  |  |  |  |  |
|  |  |  |  |  |  |  |
|  |  | % within Skin lesion | 50.00% | 50.00% |  |  |
|  |  |  |  |  |  |  |
| Abortion | No | Count | 294 | 57 |  |  |
|  |  |  |  |  |  |  |
|  |  | % within Abortion | 83.80% | 16.20% |  |  |
|  |  |  |  |  | 4.809(0.028) |  |
|  | Yes | Count | 3 | 3 |  |  |
|  |  |  |  |  |  |  |
|  |  |  |  |  |  |  |
|  |  | % within Abortion | 50.00% | 50.00% |  |  |
|  |  |  |  |  |  |  |
| Loss of | No | Count | 291 | 60 |  |  |
| Appetite |  |  |  |  |  |  |
|  |  | % within Loss of | 82.90% | 17.10% |  |  |
|  |  |  |  |  |  |  |
|  |  | Appetite |  |  | 1.233(0.267) |  |
|  | Yes | Count | 6 | 0 |  |  |
|  |  |  |  |  |  |  |
|  |  |  |  |  |  |  |
|  |  | % within Loss of | 100.00 | 0.00% |  |  |
|  |  | Appetite | % |  |  |  |
| Reduction | No | Count | 291 | 60 | 1.233(0.267) |  |
| of tail hair |  |  |  |  |  |  |
|  |  | % within Loss of | 82.90% | 17.10% |  |  |
|  |  |  |  |  |  |  |
|  |  | Appetite |  |  |  |  |
|  | Yes | Count | 6 | 0 |  |  |
|  |  |  |  |  |  |  |
|  |  | % within Loss of | 100.00 | 0.00% |  |  |
|  |  | Appetite | % |  |  |  |
| Pale eye | No | Count | 264 | 49 |  |  |
|  |  |  |  |  |  |  |
|  |  | % within Pale eye | 84.30% | 15.70% | 2.409(0.121) |  |
|  |  |  |  |  |  |  |
|  |  |  |  |  |  |  |
|  | Yes | Count | 33 | 11 |  |  |
|  |  |  |  |  |  |  |

|  |  | % within Pale eye | 75.00% | 25.00% |  |  |
| --- | --- | --- | --- | --- | --- | --- |
|  |  |  |  |  |  |  |
| Used | No | Count | 16 | 4 |  |  |
| drugs |  |  |  |  |  |  |
|  |  | % within Used drugs | 80.00% | 20.00% |  |  |
|  |  |  |  |  |  |  |
|  |  |  |  |  |  |  |
|  | Isometam | Count | 61 | 18 |  |  |
|  | idium |  |  |  |  |  |
|  |  | % within Used drugs | 77.20% | 22.80% |  |  |
|  |  |  |  |  |  |  |
|  |  |  |  |  |  |  |
|  | Diminaze | Count | 89 | 28 |  |  |
|  | ne |  |  |  |  |  |
|  |  | % within Used drugs | 76.10% | 23.90% |  |  |
|  |  |  |  |  |  |  |
|  |  |  |  |  |  |  |
|  | Isometam | Count | 53 | 2 |  |  |
|  | idium & |  |  |  |  |  |
|  |  | % within Used drugs | 96.40% | 3.60% |  |  |
|  | Diminaze |  |  |  |  |  |
|  |  |  |  |  | 28.570(0.000) |  |
|  | ne |  |  |  |  |  |
|  |  |  |  |  |  |  |
|  | Diminaze | Count | 24 | 2 |  |  |
|  | ne & |  |  |  |  |  |
|  |  | % within Used drugs | 92.30% | 7.70% |  |  |
|  | homidium |  |  |  |  |  |
|  |  |  |  |  |  |  |
|  |  |  |  |  |  |  |
|  | Homidium | Count | 10 | 6 |  |  |
|  |  |  |  |  |  |  |
|  |  | % within Used drugs | 62.50% | 37.50% |  |  |
|  |  |  |  |  |  |  |
|  | Isometam | Count | 44 | 0 |  |  |
|  | idium, |  |  |  |  |  |
|  |  | % within Used drugs | 100.00 | 0.00% |  |  |
|  | diminazen |  |  |  |  |  |
|  |  |  | % |  |  |  |
|  | e & |  |  |  |  |  |
|  |  |  |  |  |  |  |
|  | homidium |  |  |  |  |  |
| What | Treatmen | Count | 246 | 55 |  |  |
| makes you | t |  |  |  |  |  |
|  |  | % within What | 81.70% | 18.30% |  |  |
| to give |  |  |  |  |  |  |
|  |  | makes you to give |  |  |  |  |
| drug to |  |  |  |  |  |  |
|  |  | drug to animal |  |  |  |  |
| animal |  |  |  |  | 2.948(0.086) |  |
|  | Prophylaxi | Count | 51 | 5 |  |  |
|  |  |  |  |  |  |  |
|  | s |  |  |  |  |  |
|  |  | % within What | 91.10% | 8.90% |  |  |
|  |  |  |  |  |  |  |
|  |  | makes you to give |  |  |  |  |
|  |  | drug to animal |  |  |  |  |

**Table S4.** Factors that influence prevalence as determined by multilevel mixed effect modeling

|  |  |  |  |  |  |  | 95% Confidence | |  |
| --- | --- | --- | --- | --- | --- | --- | --- | --- | --- |
|  |  |  |  |  |  |  | Interval for Odds | |  |
|  |  | Coeffi | Std. |  |  | Odds | ratio | |  |
|  |  |  |  |  |  |  |  |  |  |
|  | Model Term | cient | Error | t | Sig. | ratio | Lower | Upper |  |
|  | Intercept | -3.638 | 4.1888 | -0.868 | 0.386 | 0.026 | 6.940E | 99.741 |  |
|  |  |  |  |  |  |  | -06 |  |  |
|  | Age category = (> 5 years) | 0.338 | 0.6533 | 0.517 | 0.605 | 1.402 | 0.388 | 5.070 |  |
|  | Age category = (< 5 years) | 0^b^ |  |  |  |  |  |  |  |
|  | Sex = Male | 0.489 | 0.5716 | 0.855 | 0.393 | 1.630 | 0.529 | 5.017 |  |
|  | Sex = Female | 0^b^ |  |  |  |  |  |  |  |
|  | Knowledge of AAT = Yes | -1.553 | 1.9814 | -0.784 | 0.434 | 0.212 | 0.004 | 10.432 |  |
|  | Knowledge of AAT = No | 0^b^ |  |  |  |  |  |  |  |
|  | Clinical signs = Yes | 5.758 | 0.6728 | 8.559 | 0.0001 | 316.73 | 84.314 | 1189.83 |  |
|  |  | 0^b^ |  |  |  | 2 |  | 0 |  |
|  | Clinical signs = No |  |  |  |  |  |  |  |  |
|  | UVP drugs = self-treatment | 0.285 | 1.4308 | 0.199 | 0.842 | 1.330 | 0.080 | 22.194 |  |
|  | UVP drugs = Yes | 0^b^ |  |  |  |  |  |  |  |
|  | Source=Local stores | 0.395 | 0.8460 | 0.466 | 0.641 | 1.484 | 0.281 | 7.837 |  |
|  |  |  |  |  |  |  |  |  |  |

| Source=Agrovet | 0^b^ |  |  |  |  |  |  |  |
| --- | --- | --- | --- | --- | --- | --- | --- | --- |
| Effective = Not effective | -0.342 | 1.2093 | -0.283 | 0.778 | 0.711 | 0.066 | 7.670 |  |
| Effective = Moderate | -0.189 | 1.0734 | -0.177 | 0.860 | 0.827 | 0.100 | 6.835 |  |
| Effective = Effective | 0^b^ |  |  |  |  |  |  |  |
| Technique to control AAT=Vector control, | 0.411 | 3.2197 | 0.128 | 0.899 | 1.508 | 0.003 | 849.398 |  |
| drug treatment & traditional |  |  |  |  |  |  |  |  |
| Technique to control AAT =Vector control | -0.790 | 2.8006 | -0.282 | 0.778 | 0.454 | 0.002 | 112.143 |  |
| Technique to control AAT =Drug | 0.296 | 1.1889 | 0.249 | 0.803 | 1.345 | 0.130 | 13.943 |  |
| treatment & vector control | 0^b^ |  |  |  |  |  |  |  |
| Technique to control AAT =Drug |  |  |  |  |  |  |  |  |
| treatment |  |  |  |  |  |  |  |  |
| Frequent = 9-above times/yr/animal | 0.367 | 1.4131 | 0.260 | 0.795 | 1.443 | 0.090 | 23.255 |  |
| Frequent = 4-8 times/yr/animal | -0.030 | 1.2531 | -0.024 | 0.981 | 0.971 | 0.083 | 11.420 |  |
| Frequent = 1-3 times/yr/animal | 0^b^ |  |  |  |  |  |  |  |
| Closest to National Parks game/ | -0.143 | 1.2393 | -0.116 | 0.908 | 0.866 | 0.076 | 9.919 |  |
| reserve=Not close |  |  |  |  |  |  |  |  |
| Closest to National Parks game reserve | -0.152 | 0.9944 | -0.153 | 0.879 | 0.859 | 0.121 | 6.076 |  |
| =Very close | 0^b^ |  |  |  |  |  |  |  |
| Closest to National Parks game reserve e |  |  |  |  |  |  |  |  |
| =Close |  |  |  |  |  |  |  |  |
| Farming system = Zero grazing | 0.085 | 1.2408 | 0.069 | 0.945 | 1.089 | 0.095 | 12.505 |  |
| Farming system = Communal grazing | 0^b^ |  |  |  |  |  |  |  |
| Closes to homestead=Not close | 0.022 | 0.9007 | 0.024 | 0.981 | 1.022 | 0.174 | 6.013 |  |
| Closes to homestead =Very close | 0.247 | 0.9994 | 0.247 | 0.805 | 1.280 | 0.179 | 9.145 |  |
| Closes to homestead =Close | _0_b |  |  |  |  |  |  |  |
| Fever = Yes | 1.081 | 0.8952 | 1.208 | 0.228 | 2.948 | 0.507 | 17.155 |  |
| Fever = No | _0_b |  |  |  |  |  |  |  |
| Weight loss = Yes | 0.947 | 1.2550 | 0.754 | 0.451 | 2.577 | 0.218 | 30.431 |  |
| Weight loss = No | _0_b |  |  |  |  |  |  |  |
| Skin lesion = Yes | -0.339 | 2.2105 | -0.153 | 0.878 | 0.712 | 0.009 | 55.117 |  |
| Skin lesion = No | _0_b |  |  |  |  |  |  |  |
| Abortion =Yes | 0.575 | 2.4279 | 0.237 | 0.813 | 1.777 | 0.015 | 210.914 |  |
| Abortion =No | 0^b^ |  |  |  |  |  |  |  |
| Used drugs =Isometamidium, diminazene | -0.631 | 2.8602 | -0.221 | 0.826 | 0.532 | 0.002 | 147.794 |  |
| & homidium |  |  |  |  |  |  |  |  |
| Used drugs =Homidium | -0.933 | 3.7200 | -0.251 | 0.802 | 0.393 | 0.000 | 592.776 |  |
| Used drugs =Diminazene, homidium | -0.888 | 3.1578 | -0.281 | 0.779 | 0.412 | 0.001 | 205.328 |  |
| Used drugs=Isometamidium, diminazene | -0.655 | 3.4547 | -0.190 | 0.850 | 0.520 | 0.001 | 464.741 |  |
| Used drugs=Diminazene | -0.745 | 3.2177 | -0.232 | 0.817 | 0.475 | 0.001 | 266.327 |  |
| Used drugs=Isometamidium | -0.627 | 3.1216 | -0.201 | 0.841 | 0.534 | 0.001 | 248.080 |  |
| Used drugs=No | 0^b^ |  |  |  |  |  |  |  |

UVP-Use of veterinary prescribed
